# Adolescents’ and young people’s needs and preferences for support when living with a parent with life-threatening cancer: a grounded theory study

**DOI:** 10.1186/s12904-022-01055-7

**Published:** 2022-09-22

**Authors:** Emily Bergersen, Maria Larsson, Malin Lövgren, Cecilia Olsson

**Affiliations:** 1 Karlstad University, Universitetsgatan 2, 651 88 Karlstad, Sweden; 2 Inland Norway University of Applied Sciences, Strandvegen 3, 2206 Kongsvinger, Norway; 3 Marie Cederschiöld University, Box 11189, 100 61 Stockholm, Sweden; 4 Lovisenberg Diaconal University College, Lovisenberggt. 15b, 0456 Oslo, Norway

**Keywords:** Adolescent, Cancer, Grounded theory, Palliative care, Support, Young people

## Abstract

**Background:**

Living with a parent facing life-threatening illness and losing a mom or dad at a young age can cause both short- and long-term health problems. Without satisfactory support, adolescents’ and young people are at risk of developing low self-esteem, behavioural difficulties (e.g., anger and aggression), long-term illness or premature death caused by severe mental illness, substance abuse, self-harm and suicide attempts. The aim of this study was to explore adolescents’ and young people’s needs and preferences for support as they live with a parent with life-threatening cancer.

**Methods:**

Qualitative interviews were conducted with 10 respondents (17–24 years) in Norway and Sweden. Data were analysed through grounded theory according to Charmaz.

**Results:**

Adolescents’ and young peoples’ needs and preferences for support were described through the main category ‘To feel safe and secure and to be prepared’ and further broken down into five subcategories ‘Relationships in the immediate family—balancing support and protection’; ‘The social network—support and normalcy in a carefully selected group’; ‘Maintaining everyday life—challenges in school and working life’; ‘The right support at the right time—competence, trust and continuity in meeting health care professionals’; and ‘Support outside the home—an opportunity for full transparency’.

**Conclusion:**

Adolescents’ and young peoples’ preferences for support when living with a parent facing life-threatening illness are individual and unique, but they share a common need to feel safe and secure and to be prepared.

Adolescents and young people express that they primarily want support from parents and friends, but they also want support from health care professionals, especially in situations when the ill parent becomes worse. Therefore, it is of the utmost importance for health care professionals to identify the most vulnerable adolescents and young people by mapping their social networks and paying extra attention to their needs for support when there is deterioration in the parent’s illness state. This study also highlights the importance for health care professionals to establish a good relationship with adolescents and young people to meet their needs and preferences for support. In addition, information and support are needed in a timely manner and adapted to the life-threatening ill parent’s illness state and individual’s needs and preferences to optimise preparedness.

## Background

Living with a parent facing life-threatening illness and losing a mom or dad at a young age can cause both short- and long-term health problems [1, 2]. Without satisfactory support, adolescents and young people [3] are at risk of developing low self-esteem, behavioural difficulties (e.g., anger and aggression), long-term illness or premature death caused by severe mental illness, substance abuse, self-harm and suicide attempts [4–8]. Previous research has shown that negative experiences during parental illness, death and the mourning period afterwards can increase the risk for psychosocial distress among adolescents and young people [9–11]. Age, gender, stage of cognitive and emotional development and earlier experiences are among some of the factors that will influence, in addition to the relationship between the parents and the adolescents and young people [12–16]. Furthermore, trust in healthcare professionals (HCPs) and the way they communicate and inform adolescents and young people as relatives has also been shown to have an impact [17–19].

Previous research suggests that adolescents and young people living with parents facing life-threatening illness need professional support in terms of individual and age-adapted information about diagnosis and prognosis [19–21] and that they also need emotional and social support from family and friends [22–24]. Research indicates that adolescents and young people need a respite from the parent’s illness to have the opportunity to have a normal and carefree break from being a child to a parent facing life-threatening illness [22]. In addition, previous research shows that these adolescents wish to be involved in the parents’ care and treatment on their own terms [22].

When a parent is diagnosed with cancer, HCPs play a key role in supporting adolescents, young people and their families. This responsibility for HCPs is also regulated by legislation and formal guidelines. In Sweden, the Patient Safety Act [25] states that HCPs have the responsibility to consider children’s need for information, advice and support when a parent is seriously ill or unexpectedly dies. In addition, the United Nations Convention on the Rights of the Child [26], which since January 2020 has been established as a law in Sweden, highlights children’s rights when it comes to involvement and information. In Norway, the Norwegian Health Care Act [27] states that HCPs are required to map whether adult patients have the responsibility for the care of minor children and ensure that the children receive follow-up and information when necessary. In addition, both in Norway and Sweden, it is statutory that healthcare institutions must have personnel with a special responsibility for children. The staff are required to promote work with children as relatives, inform and guide colleagues and have an overview of current support offers and help agencies [28–30], including community organisations such as “Cancer friends” https://www.cancerkompisar.se/ [https://www.cancerkompisar.se/] and “Young cancer “ https://ungcancer.se/, https://ungkreft.no/. Having responsibility for children as relatives can be an emotional task that is exacerbated by insecurity among HCPs [31]. One study indicates that some nurses, especially the novice and beginners, do not perceive that they are responsible for adolescent and young relatives [32]. It has been emphasised that HCPs want and need guidelines and research-based knowledge on how individualised support for adolescents’ and young people should be provided [31, 33]. However, it is still unclear how to identify those who need professional support and, above all, what the support should consist of and how this should be provided [10, 19, 33–36].

Living with a parent with life-threatening cancer can represent a challenging balancing act, and there is great variation in relation to the individual’s preference for support. Everyone needs and wants support when living with a life-threatening, ill parent, but this does not necessarily have to come from professionals. For some, the support they receive from friends and family is sufficient. Despite the growing trend in research aimed at adolescents’ and young peoples’ needs in relation to living with a life-threatening ill parent, there is still limited knowledge within the field. Most studies focus on grief in adolescents and young people [4, 37–40] who have already lost a parent or on the negative health outcomes [4, 6, 7, 24, 41] associated with living with a parent facing life-threatening illness. More knowledge is needed about adolescents’ and young people’s experiences of living close to their parents during the entire illness trajectory and how HCPs can provide support for them as relatives. Therefore, the aim of the current study was to explore adolescents’ and young people’s needs and preferences for support as they live with a parent with life-threatening cancer.

## Methods

### Design

The present study has a qualitative design, using constructivist grounded theory according to Charmaz [42]. The research approach is abductive, iteratively oscillating between empiricism and theory; here, knowledge is constructed in an interaction between the researcher, participants and data.

### Setting and participants

The participants were recruited through HCPs (cancer coordinators, nurses and counsellors at the psycho-oncology unit) in hospitals (*n* = 4), municipal health services (*n* = 3) and via patient and relative organisations (*n* = 3) in Norway and Sweden, both of which are considered countries where hospice-palliative care services are at a stage of advanced integration into mainstream service provision [43].

We recruited participants between 12 and 24 years old. In addition, younger siblings starting from 8 years of age and older were also offered the opportunity to participate. All participants had a parent suffering from life-threatening cancer. We recruited both those who were still living with their cancer-ill parent (biological, adoptive, step-parents or foster parents) and those who had lost their parent to cancer within the last two years. However, all the interviews focused on the period when the parent was alive. Strategic sampling was initially employed but transitioned over to theoretical sampling as our analysis progressed. Theoretical sampling was used to gather additional data based on preliminary categories in the data analysis with the intention of challenging and expanding aspects of preliminary results. All interviews (*n* = 10) were conducted digitally at a time and place chosen by the participants. A demographic presentation of the total sample is illustrated in Table 1.

**Table 1:** Demographic presentation of the participants (*n* = 10) and their life-threatening, cancer-ill parent

**Gender**	
Female	7
Male	3
**Age**	
Mean	20.7
Min-Max	17–24 years
**Nationality**	
Norwegian	3
Swedish	7
**Origin**	
Biological	10
**Family relationship**	
Daughter–mother	5
Son–mother Son–father	2 1
Daughter–father	2
**Time perspective on how long the parent has been life-threatening ill in cancer**	
Mean	3.3 years
Min-Max	6 months–12 years
**Living vs. deceased parents**	
Parents still alive	6
Deceased parent	4
**Cancer diagnosis of the ill parent**^**a**^	
Bowel cancer	1
Ovarian cancer	2
Cervical cancer	1
Brain cancer	1
Abdominal cancer	1
Breast cancer	1
Bone marrow cancer	1
Neuroendocrine cancer	1

^a^Two of the participants were siblings; hence, they had the same parent with the accompanying diagnosis

### Procedure

The information about the study was sent to HCPs, hospitals, patients, relatives and professional organisations in Norway and Sweden. The HCPs identified those who matched the inclusion criteria and passed on contact information to the researchers. The majority chose to publish the information on their websites or via social media. The next step was that adolescents and young people themselves either contacted the authors to register their interest in participation or were phoned with a request for participation from one of the authors. An information letter was sent to the adolescents and young people describing the advantages and disadvantages of participation, as well as data management. Furthermore, voluntary participation was clearly expressed by a description that they could withdraw at any time and without having to state a reason. The participants were also given the opportunity to see a counsellor after the interview if the interview provoked negative reactions and emotions, but no one expressed a need for this. Confirmation that they wanted to participate was given both in writing and orally prior to the interviews. The study was approved by ethical review boards in both Norway (37073) and Sweden (2019–06096 and 2021–02974).

### Data collection

The data collection consisted of digital individual interviews conducted from May 2020 to January 2022. The reason for the lengthy recruitment process was the COVID-19 pandemic and challenges related to contact with HCPs ‘and a lack of meeting points for adolescents’ and young people during these two years. The interviews were conducted within a safe social environment with an open and nonjudgemental atmosphere and lasted between 35 minutes and 2 hours. The Norwegian interviews were held by the first author (*n* = 3), and the Swedish interviews were held by the first and third authors (n = 3), the first and last authors (n = 3) and the third author (*n* = 1). This was done to avoid linguistic misunderstandings between the interviewer and participants. One of the authors took the lead of the interview, while the other author was more listening. However, both had the opportunity to ask questions if they desired. Having two interviewers present promoted reflection and it was often the case that the listening author picked up on aspects that the leading author had overlooked [42]. Memos were written directly after each interview in line with grounded theory. The interviews were transcribed verbatim by the first author.

The interviews were based on a thematic interview guide with topics such as the parent’s medical history (e.g., diagnosis, how long the parent had been ill, experiences related to finding out about the diagnosis), follow-up from the health care service (e.g., how they were met by HCP, what follow-up they received, what opportunities they were given for involvement, what information they were given), as well as other professional and informal supportive networks (e.g., what follow-up they received from school/work, participation in support groups or similar, social life and involvement of friends). Among other things, the participants were asked how their support should have been in a perfect world without limitations and without having to pay attention to anyone other than themselves. In line with grounded theory, the interviews were conducted as a dialogue between the participant and interviewer, where follow-up questions varied between the participants depending on how the interview developed [42]. The interview guide was slightly modified between each interview and the simultaneous ongoing analysis because the participants provided input that was interesting to explore further. The data collection took place during the COVID-19 pandemic, so statements related to this also came naturally. Participants were forced to live in isolation that affected both their social lives and their interactions with professionals, in addition to an increased concern about infecting an already seriously ill parent.

### Data management

The primary material in the research project, that is, the interviews, were processed only by the research group and stored in a secure cloud service at the university. Thereafter, in line with Swedish and European regulations, the data will be archived at the university and destroyed after 10 years.

### Data analysis

The first step of our analysis was initial coding. This was done shortly after each interview and were conducted line-by-line by the first author. To avoid putting too many interpretations in the data at an early stage, each sentence was given its code, which was closely related to the participants’ original statements [42, 44, 45]. The next step was to compress initial codes into more focused codes to have more manageable data material to work with. Finding focused codes was not a linear process but involved moving and renaming codes along the way. During this phase, mind maps and tables were created to obtain an overview of the total data material, here with the aim of finding statements that were most frequently repeated by the participants and were of interest to the aims [42, 44, 45]. The next step was to look at how the codes related to each other through theoretical coding. This process increased the abstraction of the analysis and helped in theorising the data. In addition to transcribed texts, memos were also included. Memos are an informal and spontaneous reflections of the researcher’s ideas about what was being said and what was going on and were written after each interview. Memos were also written during the data analysis to test ideas about codes and emerging theory. This contributed to a deeper level of analysis and inspired new ideas related to both codes and emerging categories. Comparisons and compositions of initial and focused codes through the constant comparative method were used in all phases of analysis and performed in collaboration with all authors. Theoretical sensitivity and reflexivity were quality assured through a previous review study [36], as well as through theoretical and practical knowledge within the research team. Simultaneous analysis and data collection continued until no new qualities were found to modify the emergent theoretical codes; hence, data saturation was reached and recruitment ended. In line with grounded theory, the results were summarised as a conceptual model that accounts for the relationships defined in the empirical data.

## Results

**Fig. 1:**
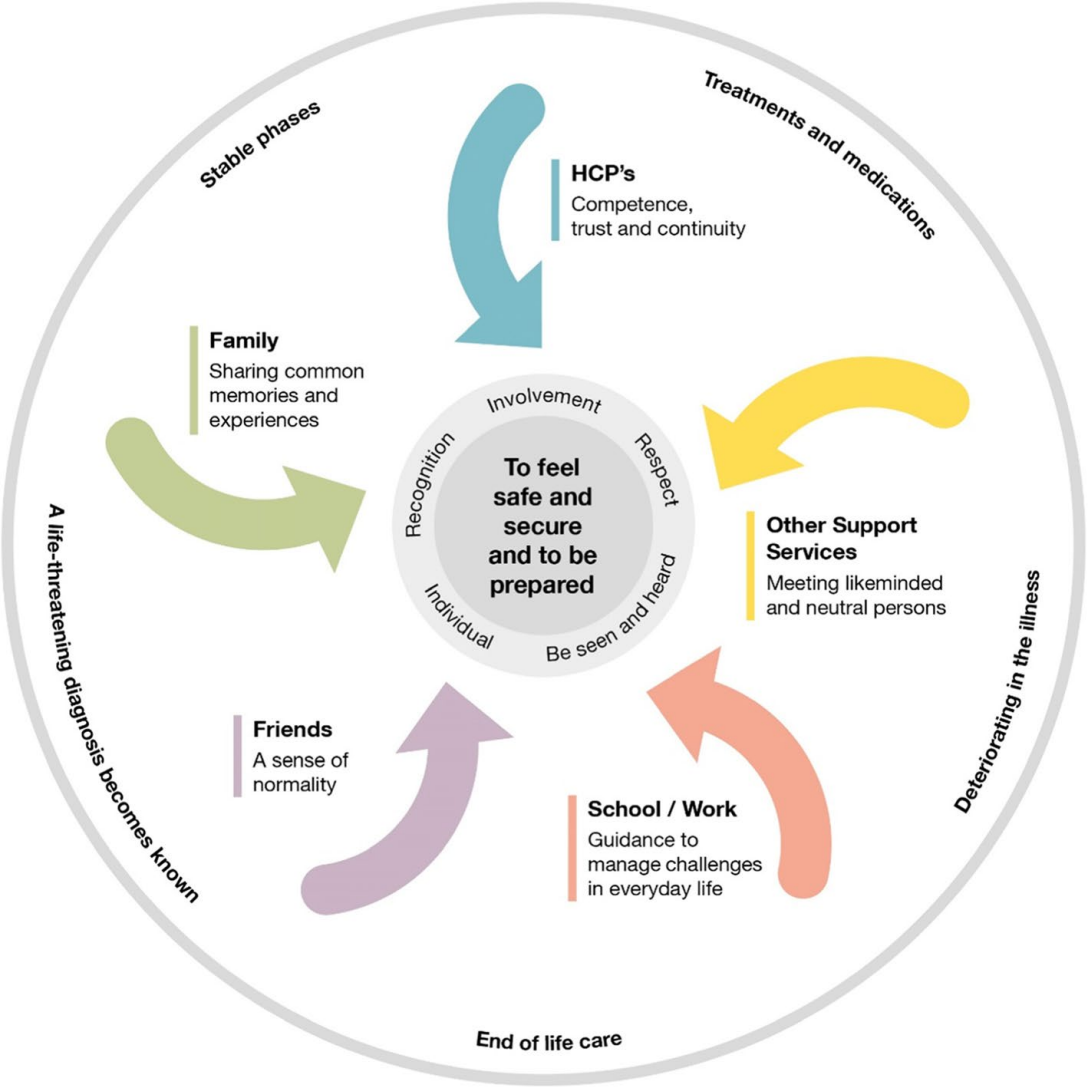
Illustration of adolescents’ and young people’s preferences for support and how these are influenced by the parents’ illness trajectory

Different resources are needed to meet the needs and preferences for support of adolescents and young people living with a parent with life-threatening cancer. Figure 1 provides a visual representation of the constructed main category, together with the five subcategories. Adolescents’ and young people’s preferences for support from families, friends, HCPs, school/work and other support services are depended on where the parent is in the illness trajectory. Meaning that adolescents and young people prefer support from different actors throughout the illness trajectory.

Subcategory 1:” Relationships in the immediate family—balancing support and protection” is constructed by preferences for sharing common memories and experiences within the family. Subcategory 2: “The social network—support and normalcy in a carefully selected group” is built up by preferences for maintaining a sense of normality. Subcategory 3:” Maintaining everyday life – challenges in school and working life” shows preferences for more guidance in managing these challenges to achieve as normal an everyday life as possible. Subcategory 4:” The right support at the right time – competence, trust and continuity in meeting HCPs” is built up by preferences for how they want to be met and supported by HCPs together with continuity of care and availability in HCPs. Subcategory 5:“Support outside the home – an opportunity for full transparency” shows preferences for meeting other adolescents and young people who are in a similar situation as well as support from neutral professionals.

The circular process is regularly ongoing throughout the illness trajectory, where all actors involved work towards adolescents and young people “To feel safe, secure and to be prepared, which constitutes the main category.

### To feel safe and secure and to be prepared

The main category explains the process of how at various stages, adolescents, young people and their networks are involved in the parent’s illness (e.g., a life-threatening illness becomes known, treatments and medications, deterioration in the illness, end-of-life care and stable phases) and how needs and preferences are met. Feeling safe was associated with being seen and heard, recognised as an individual with individual needs, involvement on their own terms and respect and openness from the family and social and professional networks they are surrounded by. If the adolescents and young people were feeling safe, they seemed to be better prepared for the different stages in the parent’s disease progression and impending or already occurring death. The preferences became visible through the adolescents’ and young people’s preferred support system and accompanying care.

### Relationships in the immediate family—balancing support and protection

The parents were the first choice of emotional and informative support for adolescents and young people and were described as unique because they shared common experiences and memories together. Spending precious and limited time with each other in general and with the ill parent in particular was highlighted by most of the participants.

*… You don’t’ want to live with a bad conscience after he (the father) dies, feeling that you did not appreciate the time. You want to feel that you did the best you could …*

All adolescents and young people stated that it was the parents who gave them illness-related information and that they could answer most things. However, many also felt that their parents were trying to protect them and that they were not always completely honest. Illness-related information could also be perceived differently by adolescents and young people and their siblings.

*... It might have been good to get information from a neutral person. Because then it is not one’s own interpretation. We are very different in how we interpret the information we get ... whether you read it positively or negatively …*

Regardless of whether she was healthy or ill, the mother was highlighted as the biggest support, while fathers were often described as more withdrawn and less open about their feelings. A common expression among the adolescents and young people was that they felt an enormous responsibility for their family members in general and the healthy parent in particular and were concerned with not being a burden. Therefore, getting help and support outside the home was mentioned by several of the interviewees.

### The social network—support and normalcy in a carefully selected group

Most of the adolescents and young people pointed out that friends provided both social and emotional support during the parent’s illness because they represented a more normal part of their lives, both at school and in their free time. However, the majority had only informed their closest friends whom they knew they could trust and who could endure hearing the truth about the extent of the parent’s illness, without withdrawing from the situation. At the same time, they also wanted to have fun with friends, not always talking about the illness. Many were also concerned about not being a burden to their friends, and some did not want their friends to know anything at all.

*... I consider being with my friends as ... it's probably a free zone. I do not want them to know! ...*

Some of the adolescents and young people also wanted friends and other peers to show more consideration for them and their situation instead of talking about trivial problems that they found provocative. Most stated that the parents’ illness had given them a new perspective on life, forcing them to grow up prematurely and that it was no longer as easy to feel belonging to peers.

*... They (older sisters) had a place to live, and they had their partners and such. And I have none of that! So I think I became more lonely ...*

The social network of friends was important for adolescents and young people because they often lacked other contact persons, such as a spouse or colleague, on whom adults often rely.

### Maintaining everyday life – challenges in school and working life

Some adolescents and young people went to school, while others worked. The school was described as an important arena in which they had felt well-taken care of by the teachers. At the same time, there were several who had experienced the school as unresponsive, with little or no follow-up by the school health service. The transition from upper secondary school to higher education was experienced as challenging, and more facilitation was desired.

.*... No one (at the university) has, what I can think of, approached me and asked if I wanted to talk to a social worker or something. At all! Maybe it's because I'm a young adult .... When I went (to high school) last year ... the principal and the teachers were there, I got to talk to the social worker immediately ... the school contributed to a lot of support ...*

Some struggled with moving away from home during the parents’ illness, while others struggled with motivation and chose to discontinue their studies. More guidance related to manage school and everyday life was mentioned by several participants. Those who worked also asked for more support from employers.

*… You have not encountered such things when you are 23 years old ... and you have no idea what to do ... when the employer does not contribute! Just helping with the practical ...*

Several had experienced a lack of understanding and little facilitation regarding practical tasks such as changing work shifts, applying for sick leave and follow-up when returning to the workplace. The experience of being treated like an adult but not feeling like one was described as overwhelming.

### The right support at the right time—competence, trust and continuity in meeting HCPs

All the adolescents and young people stated that they needed informative support from HCPs from time to time, often in conjunction with major changes in the parent, such as starting a new treatment. However, there were individual differences; some wanted information earlier in the process to feel prepared, while others preferred timely information only when needed.

*... It's probably better if the support comes at a later stage ... then, you may have managed to breathe a little ...*

One factor that was important to many was that HCPs should have competence in cancer, palliative care (including death and dying) and how to handle young relatives because they perceive and process information differently than adults. It was important for them that HCPs could respond to things that were directly related to the ill parent and that they received information about medication, treatment and prognosis in a way that was easy to understand while having an opportunity to ask follow-up questions when needed.

*... it’s very important that HCPs have information! They actually have to know things ... to calm people down. Having information to support the individual ...*

The adolescents and young people appreciated having a few HCPs to relate to as they became more confident that HCPs had relevant insight into the parent’s medical history, in addition to making it easier by not having to repeat themselves. Trust was also associated with HCPs doing everything they could for the parent and alleviating problems such as pain relief and preventing infections (COVID-19), as well as treating the parent with respect. Trust could also be associated with availability and knowing that HCPs came when they were called.

*… It felt safe having the doctor and the nurses nearby who we could call at any time ... they came regularly …*

The adolescents and young people described a positive experience of HCPs’ treatment and care of the ill parent; at the same time, the majority lacked HCPs’ care of themselves as relatives. They stated that HCPs should contact them directly as part of a mandatory follow-up of young relatives; instead, they were urged to contact HCPs when needed, but this was a big step to take.

*... I really think that HCPs should say, ‘Your mom is ill. Do you want someone to tell you more about it?’ Then, my answer would have been ‘YES’ without thinking about it! ... but when I do not know about the possibility, you sit there and are a little lost in a way …*

The few who did experience support from HCPs experienced that the contact disappeared more or less directly after the parent died. Finding where support was available for adolescents and young people, both in real life and on the internet, was described as needed, and those who had this support found it by chance or through resourceful family members.

### Support outside the home—an opportunity for full transparency

In relation to preventing loneliness, seeking support groups was a positive experience for some. However, it is important to meet someone who matches oneself regarding age, gender and the parent’s diagnosis and stage of development as much as possible, as well as meeting someone who is concerned with the same things as oneself. Those who had parents with rarer forms of cancer seemed to have greater challenges in meeting like-minded people. Some had also experienced that it was easier to find support groups for those who had already lost their parent than for those who still had the parent alive.

*… I remember that it was a group for those who had lost a parent ... but they obviously had a requirement that ... it was like nine months since the parent had passed away. I said I have a very sick dad and he may die this spring ... but they said; no, it’s nine months that applies anyway …*

Several of the adolescents and young people called for the opportunity to talk to what they called ‘a neutral person’, such as a social worker who was not emotionally involved in the family. However, for these conversations to be helpful, personal chemistry with the counsellor had to be present and the adolescents and young people had to talk freely and on their own terms about what bothered them, without being analysed. Furthermore, the social worker had to have some background history and be comfortable talking about serious illness and death.

*... It did not feel well talking to the social worker. But it was mostly because that ... it was Corona and a video call. And it felt so strange to sit in my room and talk to a counsellor about my mother’s cancer diagnosis ...*

During the COVID-19 pandemic, there were several who had digital meetings with social workers, something most had not appreciated because it was too impersonal and they had to be conscious of what they were saying when they were at home with the family. However, geographical proximity to support services is nevertheless important.

## Discussion

The aim of the current study was to explore adolescents’ and young people’s needs and preferences for support as they live with a parent with life-threatening cancer. As reported elsewhere, their preferences for support are unique and individual, but with a common need to feel safe and secure and be prepared. The results indicate that the need and preference for support is best met through HCPs establishing a good relationship with all members of the family. Although the adolescents and young people expressed that they primarily wanted support from parents and then from friends, HCPs must take their responsibility towards young adults as relatives to a parent facing life-threatening illness. To identify the most vulnerable adolescents and young people in need of support, they need to map their social networks. In addition, informative support is needed, here in a timely manner and adapted to the parent’s disease trajectory and illness state, to give adolescents and young people a higher level of preparedness. Preparedness is a term that is increasingly used in palliative care and is described as something valuable, especially in connection with the relief of stress and discomfort in family caregivers [46–49]. The concept is aimed at the caregiver’s role and readiness and includes fulfilling the physical and emotional requirements of the patient, planning care and managing the stressors [50, 51].

The adolescents and young people in the current study preferred emotional and informative support from their parents. However, they experienced that their parents had some limitations in what they could respond to regarding illness-related information and that they were not always completely honest with them. Previous research shows that parents have called for a more individualised approach from HCPs to their children (regardless of age) and that they themselves need repeated support and advice to maintain their parental responsibilities [52]. Parents often spend a lot of energy protecting their children. Adolescents and young people need to be included, prepared and given the opportunity to express emotions in a safe environment [11, 53, 54]. By HCPs acknowledging adolescents’ and young people’s needs and supporting them, it will also be possible to support the parents so that they have the strength to hold the parental role [55, 56]. However, HCPs tend to find it challenging to talk to adolescents and young people because they want to protect them and often believe that they are either too young to understand or not able to cope with the fact that their parent is dying [57].

Adolescents and young people rely on a complex network of different supporting actors. A supportive network can contribute to a well-functioning everyday life, while a lack of this can contribute to even more vulnerability among adolescents and young people. Previous research shows that adolescents and young people have a great need to feel a sense of belonging to friends but that living with a parent facing life-threatening illness can affect their ability to build relationships. Adolescents and young people undergo changes in values that make them no longer able to experience belonging to peers, and the parent’s illness makes it difficult to have visits at home [58, 59]. After a parent dies, adolescents and young people may have a fundamental fear of risking losing someone again, so they choose to isolate themselves and become very alone in their grieving process. Social isolation in adolescents and young people can increase the risk of depression, suicide attempts and low self-esteem [60].

The results of the current study show that adolescents and young people want greater involvement from HCPs in general and increased support related to information in particular, which is consistent with previous research [61–65]. Furthermore, adolescents and young people express a need to be seen and acknowledged by HCPs; they should be met with compassion, in addition to professional competence, which is also supported by recent research [65]. However, HCPs face several barriers in this regard. They may experience low self-esteem related to their own competence and want more knowledge about developmental stages and communication with young relatives [32, 66–69]. One proposal to overcome these barriers is to offer HCPs more education in meeting adolescents and young people as relatives, preferably in combination with simulation exercises where HCPs get the opportunity to develop their practical as well as theoretical skills.

To prevent and counteract serious health challenges in adolescents and young people, it is important to map the individual’s preferences and needs for support, as well as their support network. The lack of suitable measuring instruments for this purpose has also been highlighted in previous studies [36, 70, 71]. There is, however, an instrument called “the Offspring Cancer Needs Instrument” (OCNI) [72], which is aimed to assess psychosocial unmet needs of young people who have a parent with cancer. An instrument that is more based on preferences in addition to unmet needs is requested. One of the most important contributions of the present study is that adolescents’ and young people’s preferences and needs must be taken care of throughout the parent’s illness trajectory and that different stages of the illness require different approaches. From the perspective of adult caregivers, studies have been conducted on different transitions in the illness trajectory during which relatives must adapt to a new role for each phase of the illness. Furthermore, being able to understand these transitions has been highlighted as an important part of supporting relatives [73, 74]. The transition process is unique to the individual and can be complex because it requires new knowledge, changed behaviour, a supportive environment and the re-examination of one’s self-image [75, 76]. Previous research indicates that it is in conjunction with ‘tipping points’ in the parent’s disease trajectory that young relatives are the most vulnerable and that HCPs should be aware of this when providing support [36]. In addition, these transitions must be balanced with normal cognitive, physical and emotional changes in adolescents and young people, which in and of itself can be demanding.

### Methodological considerations

According to Charmaz, the validity and reliability of a grounded theory study are determined by credibility, originality, resonance and usefulness [42].

The credibility was strengthened by a structured approach for conducting the interviews and processing the data [77]. The interviews were conducted in secure digital rooms where the only access was for the interviewers and participants. Accurate transcripts were ensured through high-quality image and sound transmission. Memos were written, and the interviews were transcribed verbatim to ensure that all statements were carefully documented. Each step in the analysis process is documented in the form of text, tables and drafts of figures. Coding was conducted by the first author and then discussed and modified with the other three authors. Constant comparisons between the data and categories ensured a logical connection and that the categories would be grounded in the data.

The originality of the current study is reflected in an extended understanding of how HCPs can meet adolescents and young people living with a parent facing life-threatening illness. Further, it could be considered as offering new insights because there are few previous studies in this context.

The resonance of the study was strengthened by having two researchers present for most of the interviews, where one of the interviewers’ roles was to summarise statements and allow the participant to confirm or disagree with our understanding of what was said [78, 79]. The same was applied to linguistic barriers, where one of the interviewers always had the respondent’s language as her first language. Furthermore, the resonance was strengthened through several researchers asking questions of the same text, challenging existing preunderstandings and, in this way, contributing to data interpretation [77, 79]. There was some variation related to the parent’s illness trajectory among the participants. This seemed to somewhat influence their opinions because those who had already lost a parent appeared more reflective on what they had missed in terms of support, while those who still lived with a parent facing life-threatening illness were mostly focused on support given to the parent and the rest of the family. Therefore, a weakness of the current study may be that not all the participants were able to fully express their individual preferences and need for support. The study also has limitations related to adolescents and young people with a non-biological origin because all participants had a biological affiliation to their parent facing life-threatening illness. However, we have succeeded in including participants living in both urban and rural areas.

Strategic sampling was initially employed but transitioned over to theoretical sampling as our analysis progressed. By studying a more homogeneous group with less dispersion in maturity, transferability increased. However, studying younger adolescents and their needs and preferences for support is nevertheless important. The results from the present study could also be useful to adolescents and young people (aged 17–24) living with parents facing diagnoses other than cancer. Furthermore, the study could have limited transferability because it was conducted in Norway and Sweden, which both have well-developed health services, which may not be the case in many other countries.

## Conclusion

The results of the current study show that adolescents and young people want to feel safe, secure and prepared when living with a parent facing life-threatening illness and that their needs and preferences for support change as the parent’s illness progresses. Adolescents and young people seek support in their own network and prefer support from parents and friends. Therefore, it is important to identify those with a weak social network because they are potentially more vulnerable. However, previous research suggests that parents experience it as challenging to care for adolescents and young people when illness affects the family. Hence, more research focusing on how the family handles illness, communication and support is recommended.

Although most of the participants wanted informative, emotional and social support from friends and their parents, there is still a large amount of desire for professional support. Adolescents and young people call for more informative and emotional support from other support services in general and from HCPs in particular. Most experience that a lot of responsibility is placed on them, so they demand more initiative from professionals. As a means to facilitate communication between HCPs and adolescents and young people to identify their needs, it is of interest to test the generated model in the current study using an intervention approach. The model could be used as a mapping tool for HCP to identify young relatives’ supporting networks, preferences and needs. The overriding purpose of this would be to ensure that those who are most in need of support are identified and to further ensure that they receive more individual follow-up.

## Data Availability

The datasets generated and/or analysed during the current study are not publicly available due to individual privacy could be compromised but are available from the corresponding author on reasonable request.

## Abbreviations

HCPs: Healthcare professionals
